# Image quality and attenuation values of multi detector CT coronary angiography using high iodine-concentration contrast material: A comparison of the use of iopromide 370 and iomeprol 400

**DOI:** 10.3109/02841851.2010.509740

**Published:** 2010-09-16

**Authors:** Eun Young Kim, Dae Wook Yeh, Yeon Hyeon Choe, Won Jae Lee, Hyo Keun Lim

**Affiliations:** 1 Department of Radiology and Cardiovascular Imaging Center, Samsung Medical Center, Sungkyunkwan University School of Medicine, Seoul, Republic of Korea; 2 Department of Radiology, Gachon University Gil Hospital, Incheon, Republic of Korea

**Keywords:** Coronary arteries, CT angiography, contrast agent

## Abstract

**Background:** Effects of high iodine-concentration contrast material on the image quality of coronary CT angiography (CCTA) have not been well evaluated.

**Purpose:** To compare the image quality and attenuation values of CCTA between patients administered iopromide 370 and iomeprol 400 with the use of 64-slice multidetector CT. **Material and Methods:** Patients were prospectively enrolled and were randomized into two groups (group A, 151 patients received iopromide 370, iodine flux = 1.48 g I/s; group B, 146 patients received iomeprol 400, iodine flux = 1.60 g I/s). CT attenuation was measured in the coronary arteries and great arteries and measurements were standardized based on an iodine flux of 1.5 0 g I/s. The image quality of 15 coronary artery segments was graded by two radiologists in consensus with the use of a four-point scale (1 = excellent to 4 = poor enhancement). Non-parametric statistical approaches were used to compare the two groups.

**Results:** The median attenuation values in the coronary arteries were 454 HU and 464 HU for iopromide 370 and iomeprol 400, respectively, and they did not differ (*P* = 0.26). When standardizing for an iodine flux, significantly higher attenuation values were found for iopromide 370 (median = 460 HU, range = 216-791 HU) compared with iomeprol 400 (median = 435 HU, range = 195—758 HU) (*P* = 0.006). The median image quality score of coronary arterial segments was 1 (range 1—2) for both groups (*P* = 0.84).

**Conclusion:** The attenuation values in the coronary arteries after injection of the same amount of two high iodine-concentration contrast materials at the same flow rate with different iodine fluxes are similar with no difference in image quality. With standardization for an iodine flux, the attenuation is significantly higher when using iopromide 370.

The recent development of multidetector CT (MDCT) has enabled noninvasive imaging of the coronary arteries. Because of rapid cardiac motion, high temporal resolution is essential for cardiac CT techniques and spatial resolution should be suffi cient for depiction of the branches of coronary arteries. In addition, optimal enhancement is essential for the entire diagnostic process and all diagnostic images including curved multiplanar reformation and volume-rendering images for coronary arteries to ensure reliable results during image post-processing. Recent studies have described the use of contrast material with high iodine content (370 mg I/ml or 400 mg I/ml) for coronary CT angiography (CCTA) (1–4). However, the effects of high iodineconcentration contrast material on the vessel visibility of CCTA using a 64-slice MDCT have not been well evaluated.

The purpose of this prospective study was to compare the image quality of CCTA using a 64-slice MDCT with two contrast agents with high iodine concentrations (iopromide 370 mg I/ml and iomeprol 400 mg I/ml). The attenuation obtained in the coronary arteries and the great arteries as well as the subjective degree of enhancement in the coronary arterial segments were evaluated.

## Material and Methods

### Patient population

From August 2007 to December 2008, 337 consecutive patients (206 men and 131 women; mean age, 54 ± 10 years; age range, 22–75 years) referred for CCTA for suspected coronary artery diseases were enrolled pro-spectively in the study. Patients who had arrhythmia, renal insufficiency (a serum creatinine level more than 1.5 mg/dl), a history of allergic reaction to contrast material, previous history of surgery or stenting for coronary artery diseases, heart failure, and women who were potentially pregnant or nursing were not eligible for study participation. Patients who were unable to cooperate with breath-holding for at least 10 s or had a body weight above 90 kg or below 40 kg were not enrolled, to limit the heterogeneity within the patient population. The institutional review board approved this study and all patients provided written informed consent to participate in this study. Images of the study were excluded from the analysis in the presence of poor image quality caused by a severe motion artifact, extensive calcification or inappropriate scan coverage.

Patients were randomly assigned into two groups by the use of permuted block randomization (5), which differed with regard to the iodine concentration of the contrast agent that was administered. Group A patients received iopromide 370 (370 mg I/ml, Ultravist 370; Bayer Schering Pharma, Berlin, Germany) and group B patients received iomeprol 400 (400 mg I/ml, Iomeron 400; Bracco Imaging, Milan, Italy). For each patient, age, sex, height, and body weight were recorded.

### MDCT

Coronary CT angiography was performed on a 64-row detector system (Aquilion 64, Toshiba Medical Systems, Otawara, Japan) in the craniocaudal direction to cover from the aortic root to the caudal end of the heart. Before an examination, all patients were instructed to take a deep breath and to hold their breath. Patients with a prescanning heart rate of 65 beats per minute or higher were given 100 mg of metoprolol (Seloken; AstraZeneca, Zoetermeer, The Netherlands) orally 1 hour before CT scanning. Just before the injection of contrast material, 0.6 mg nitroglycerin was administered sublingually for vessel dilation.

The contrast agents were prepared at 37°C and were injected with an 18-guage needle through the right antecubital veins by the use of a dual-syringe power injector (Stellant-Dual Flow; Medrad, Pittsburgh, Pa., USA). Contrast agents were administered at a rate of 4 ml/s and were followed by a 40 ml saline flush at the same rate (6). The resulting contrast material volume and injection rate, respectively, were 70 ml and 4 ml/s (total injection time, 17.5 s) for all patients. This procedure reflected a clinical routine that resulted in an iodine flux (iodine delivery rate, IDR) of 1.48 g I/s for iopromide 370 and 1.60 g I/s for iomeprol 400. The IDR was calculated as follows: IDR (g I/s) = [iodine concentration (mg I/ml) X contrast flow (m1/s)]/1000 mg/g (7). In the analysis, attenuation values in the vessels of the two groups were additionally standardized on a flux of 1.50 g I/s for the comparison of the attenuation values as described in the statistics section.

Synchronization between the passage of contrast material and data acquisition was achieved with the use of a real-time bolus tracking technique (SureStart; Toshiba Medical Systems, Tokyo, Japan) using a region of interest (ROI) positioned in the ascending aorta. The trigger threshold inside the ROI was set at 200 HU. The main scanning parameters were as follows: number of detectors, 64; individual detector width, 0.5 mm; gantry rotation time, 400 ms; tube voltage, 120 kVp; tube current, 400 mA; feed/rotation, 3.2 mm; feed/second, 8.0 mm. A phantom of the American Association of Physicists in Medicine (AAPM) was used for the calibration of the CT scanner to ensure reproducible measurement of CT ROI at a 6-month interval. An acceptable limit of the attenuation of water was 0 ± 4 HU. For daily quality control of CT, air calibration was used.

### Image data reconstruction

Data collection and analysis were performed using previously reported methodology (2, 8) with some modifications. The data set was reconstructed with retrospective electrocardiography gating with time windows of 70%, 75%, and 80% of the R-R interval for one of the mid-diastolic phases of cardiac cycles to represent motion-free images. For patients with a heart rate of more than 70 beats per minute during scanning, additional reconstruction of images for systolic phases was performed. For patients who showed suboptimal image quality on predetermined phases due to variable or high heart rates, the best phase for image reconstruction was selected after a review of multiphase images of one slice at the mid-heart level by 10 ms or 1% interval throughout the cardiac cycle.

With the use of axial data, an experienced radiologist reconstructed the three-dimensional volume-rendered images and curved multiplanar reformation images of the coronary arteries using commercial software (Aquaris ver. 3.5.2.1; TeraRecon, SanMateo, Calif., USA).

### Evaluation of contrast enhancement in the coronary arteries

**Figure 1:**
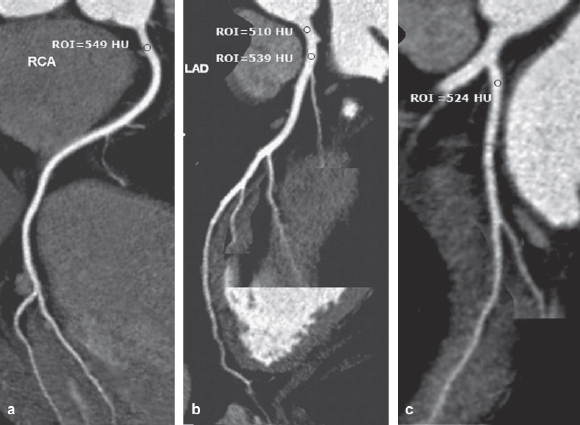
Curved planar reformation images of coronary CT angiography using iomeprol 400 are shown in a 47-year-old male. The attenuation numbers using the region of interest technique of the proximal portion of the coronary arteries were higher than 500 HU for the proximal right coronary artery (a), the left main coronary artery and the proximal left anterior descending branch (b), the proximal left circumflex artery (c). On subjective analysis, 13 segments of the coronary arteries were assessed as grade 1 (posterolateral and posterior descending branch of left circumflex artery were not found).

Coronary CT angiography was analyzed by consensus of two experienced cardiac radiologists who were blinded to contrast material used. Oblique coronal or sagittal sections in the dataset were selected to measure the attenuation value objectively using an ROI placed at the proximal part of the four main coronary arteries (right coronary artery, RCA; left main artery, LM; left anterior descending artery, LAD; and left circumflex artery, LCX) (6). The ROIs were drawn as large as possible within the vessels with care taken to avoid motion artifacts as well as calcification or soft plaques (Fig. 1). The overall visualized length without motion artifact of each coronary artery depicted on a curved planar reformation image was extracted by the use of the standard software and compared for both groups.

The readers subjectively evaluated the image quality based on a 15-segment American Heart Association (AHA) model (9). The proximal, middle, and distal segments of RCA (segments 1,2, 3), posterior descending branch of RCA (segment 4), LM (segment 5), and the proximal, middle, and distal segments of the LAD (segments 6, 7, 8), the first and second diagonal vessels (segments 9, 10), proximal and distal LCX (segments 11, 13), obtuse marginal branch (segment 12), and posterolateral and posterior descending branch of LCX (segments 14,15). For each segment, image quality was graded with the use of a four-point scale. Scores were defined as grade 1 for excellent (strong homogeneous enhancement with sharply defined vessel edges), grade 2 for good (homogeneous enhancement with mildly blurred vessel edges), grade 3 for fair (inhomogeneous enhancement with moderately blurred vessel edges), and grade 4 for poor (inhomogeneous enhancement with markedly blurred vessel edges) image quality. Grades 1, 2, and 3 were assumed as scores of diagnostic image quality. If the segment was not delineated by the field of view for its hypoplasia or aplasia, it was counted as “not applicable".

### Evaluation of contrast enhancement in the great arteries

**Figure 2:**
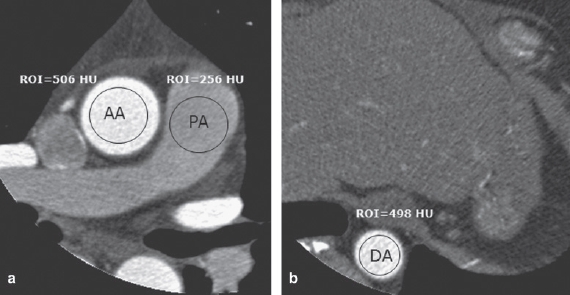
Axial images of coronary CT angiography in the same patient as in Fig. 1 are shown. The attenuation numbers of the great arteries were 506 HU for the ascending aorta (AA), 256 HU for the main pulmonary artery (PA) (a), and 498 HU for the descending aorta (DA) at the level of the inferior margin of the heart as determined on axial images (b). The calculated consistency of contrast material was 8 HU.

The CT attenuation of both groups was measured objectively using the ROI technique at the proximal ascending aorta, main pulmonary artery, and distal descending thoracic aorta at the level of the inferior margin of the heart on axial images (Fig. 2). Consistency of contrast enhancement was also assessed by calculation of ROI differences as follows: consistency of contrast enhancement = (attenuation of proximal ascending aorta — attenuation of distal thoracic aorta).

### Evaluation of streak artifacts in the right atrium

Artifacts due to insufficient mixing of contrast material in the right atrium were assessed using a four-point scale. Scores were defined as grade 1 for no streak artifact, grade 2 for a mild streak artifact without an obscured vessel segment, grade 3 for a moderate streak artifact with a mild but acceptable degree of vessel segment obscuration, and grade 4 for a severe streak artifact with markedly obscured vessel segments.

### Evaluation of adverse effects due to contrast material

Vital signs including heart rate were monitored before the injection of contrast material and during CCTA. All types of adverse effects of contrast agents were also recorded and vital signs were closely monitored during CT examinations.

### Statistical analysis

Sample size was calculated by assuming that the grades of image quality in each coronary segment in two groups would not be different. If one supposes that the proportion of grade 1 would be 90% in each group and that difference in proportions of grade 1 in the two groups less than 10% would be regarded as no difference in them, then one needs 220 patients with 80% power and 5% type I error.

Demographic data such as sex, age, body mass index (BMI), height, baseline heart rate, and patients’ characteristics were compared using the chi-squared test and Wilcoxon two-sample test as appropriate. The attenuation values of the coronary arteries and the great arteries and the visualized length of each coronary artery without motion artifact were averaged for all patients in each group and the overall average was used to compare the two groups using Wilcoxon rank sum tests for single vessel and *t* test on ranks based on a mixed linear model to account for the correlation of vessels within the same patient (coronary arteries only). All tests were based on ranks as deviances from normality were expected for the attenuation values. In addition, the attenuation values were standardized for an iodine flux of 1.50 g I/s by dividing the attenuation values by the iodine flux used and by multiplying by 1.50 (10). These were analyzed in the same way as for the raw attenuation values. The results of subjective analysis for the image quality of 15 coronary arterial segments graded with the use of the four-point scale and streak artifacts of the right atrium were assessed by the use of a multinomial regression based on generalized estimating equations (GEEs) taking into account multiple vessels within the same patient. Independence was used as working correlation matrix. The number of patients who showed adverse reactions to contrast agents was assessed using the chi-square test. All tests were performed two-sided; *P* values <0.05 were regarded as statistically significant. Data processing and analysis were performed with SPSS (version 10.0; SPSS, Chicago, IL, USA) and SAS (version 9.2, SAS Institute, Cary, NC, USA).

## Results

### Demographic data

Among 337 patients initially recruited for this study, 297 patients (151 patients in group A and 146 patients in group B) were ultimately included in this investigation. Forty patients were excluded from the analysis because of poor image quality caused by a severe motion artifact (*n* = 11), extensive calcification (*n* = 27) or inappropriate scan coverage (*n* = 2). There were 175 men and 122 women (age range, 22—75 years; median age, 54 ± 10 years). Patients’ demographics and characteristics were not significantly different between the two groups in terms of sex, age, BMI, height, baseline heart rate, use of premedication, and symptoms except atypical chest pain (Table 1).

**Table 1:** Demographics and baseline characteristics.

Characteristics	Iopromide 370	Iomeprol 400	Comparison, *P* value
No. of patients	151	146	
No. male (%)	87 (58%)	88 (60%)	0.64†
Age (years)*	55 ± 9 (22–75)	52 ± 11(22–75)	0.07‡
Height (cm)*	164 ± 8(145–188)	166 ± 8(148–184)	0.26‡
BMI (kg/m^2^)*	24.6 ± 3.0 (18.4–37.0)	24.8 ± 2.7 (15.8–32.3)	0.35‡
Heart rate (beats per minute)*	70 ± 11(50–106)	71 ± 11(42–102)	0.24‡
Hypertension	44 (29%)	45 (31%)	0.75
Diabetes	12 (8%)	17 (12%)	0.28
Smoking	25 (17%)	36 (25%)	0.08
Exertional chest pain	22 (15%)	16(11%)	0.35
Stable chest pain	35 (23%)	36 (25%)	0.77
Atypical chest pain	26 (17%)	13 (9%)	0.03
Hyperlipidemia	22 (15%)	19 (13%)	0.70
Use of metoprolol	87 (58%)	96 (66%)	0.15
Use of nitroglycerin	116(77%)	104 (71%)	0.27

* Data are mean values with standard deviations, and numbers in parentheses are ranges.

† Two-group chi-squared test (two-sided).

‡ Two-group Wilcoxon rank sum test (two-sided).

### Evaluation of contrast enhancement in the coronary arteries

**Figure 3:**
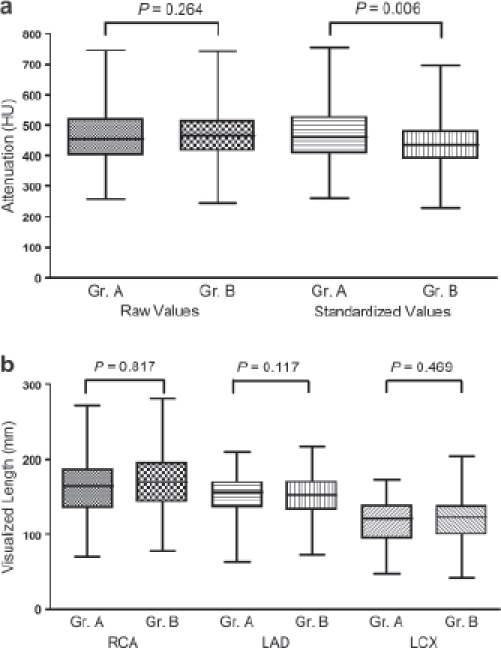
(a) Boxplot of raw and standardized attenuation values for enhanced coronary vessels with *P* values for the comparison of groups. The raw attenuation values in the coronary arteries showed no significant difference between two groups with the median attenuation value of 454 HU (range 213-780 HU) for iopromide 370 (group A, Gr.A) and that of 464 HU (range, 208–809 HU) for iomeprol 400 (group B, Gr.B), respectively (*P* = 0.26). After standardization with an iodine flux of 1.5 g I/s, the attenuation using iopromide 370 was significantly higher in the coronary arteries (except LAD, *P* = 0.0539). (b) Boxplot of visualized length of coronary vessels along with *P* values for the comparison of groups. The measurements were similar in both groups.

No differences were found between the median attenuation values (454 HU for iopromide 370 and 464 HU for iomeprol 400, *P* = 0.26) in the coronary arteries in the two groups without standardization for an iodine reflux (Table 2). After standardization for an iodine flux of 1.5 g I/s, the median attenuation value for iopromide 370 was higher than that for iomeprol 400 (460 HU and 435 HU, respectively, *P* = 0.006) (Table 2, Fig. 3a). No significant effect of the location of vessel was found on the attenuation values (*P* = 0.99 and *P* = 0.99 for raw and standardized values, respectively). The median visualized length of the RCA, LAD, and LCX depicted on curved planar reformation images was not significantly different between the two groups (*P* > 0.05, each) (Table 3, Fig. 3b). A significant effect of the location of vessel was found on the visualized lengths of the three coronary arteries in each group (*P* < 0.0001).

**Table 2:** Attenuation in the proximal segments of the four coronary arteries.

Vessel	Iopromide 370	Iomeprol 400	Comparison, *P* value
RCA (HU)	457 (219–780)	472 (208–809)	0.2533†
n_A_ = 151, n_B_ = 146	*463 (222–791)	*442 (195–758)	0.0245 *†
LM (HU)	454 (271–746)	455 (241–677)	0.4756†
n_A_ = 151, n_B_ = 146	*460 (275–756)	*427 (226–635)	0.0022 *†
LAD (HU)	459 (213–778)	472 (236–757)	0.1031†
n_A_ = 151, n_B_ = 146	*465 (216–789)	*443 (221–710)	0.0539 *†
LCX (HU)	449 (280–716)	457 (252–745)	0.5194†
n_A_ = 151, n_B_ = 146	*455 (284–726)	*428 (236–698)	0.0038 *†
Average	454 (213–780)	464 (208–809)	0.2644‡
n_A_ = 604, n_B_ = 584	*460 (216–791)	*435 (195–758)	0.0060 *‡

Data are medians and ranges in parentheses. n_A_ = number of vessels in group A (iopromide 370); n_B_ = number of vessels in group B (iomeprol 400).

* Attenuation in the proximal segments of the four coronary arteries standardized with an iodine delivery rate of 1.5 g I/s.

† Two-group Wilcoxon rank sum test (two-sided).

‡ *t* test (two-sided) on ranks based on a linear mixed model.

**Table 3:** Visualized length of three coronary arteries.

Vessel	Iopromide 370	Iomeprol 400	Comparison, *P* value
RCA (mm) n_A_ = 151, n_B_ = 146	121 (47 – 173)	123 (42 – 204)	0.8172*
LAD (mm) n_A_ = 151, n_B_ = 146	167 (70 – 271)	172 (77 – 282)	0.1165*
LCX (mm) n_A_ = 151, n_B_ = 146	157 (64 – 209)	152 (72 – 218)	0.4694*
Average n_A_ = 604, n_B_ = 584	147 (47 – 271)	148 (42 – 282)	0.2005†

Data are medians and ranges in parentheses. n_A_ = number of vessels in group A (iopromide 370); n_B_ = number of vessels in group B (iomeprol 400).

* Two-group Wilcoxon rank sum test (two-sided).

† *t* test (two-sided) on ranks based on a linear mixed model.

A total of 3602 segments of 297 patients were evalu-able for subjective analysis of contrast enhancement, excluding 853 segments for their hypoplasia or aplasia. In a subjective assessment with the use of a four-point scale, each segment of the coronary arteries showed excellent or good contrast enhancement. For iopromide 370, 91.8% (*n* = 1699) of 1851 coronary segments had a score of grade 1 and the remaining 8.2% (*n* = 152) had a score of grade 2. For iomeprol 400, 91.6% (*n* = 1604) of 1751 coronary segments had a score of grade 1 and the remaining 8.4% (*n* = 147) had a score of grade 2. There was no significant difference for the image quality score of each coronary segment between the two groups (*P* = 0.84).

### Evaluation of contrast enhancement in the great arteries

The median attenuation values of the proximal ascending aorta, main pulmonary artery, and descending thoracic aorta were not different between the two groups (Table 4). After standardization for an iodine flux of 1.5 g I/s, the median attenuation of proximal ascending aorta and descending thoracic aorta were significantly higher for iopromide 370 than for iomeprol 400.

**Table 4:** Attenuation in the great arteries.

Vessel	Iopromide 370	Iomeprol 400	Comparison, *P* value
Proximal ascending aorta	447 (156–656)	444 (254–682)	0.2341†
n_A_ = 151, n_B_ = 146	*453 (158–665)	*416 (238–639)	0.0164*†
Main pulmonary artery	267 (101–674)	270 (110–714)	0.9291†
n_A_ = 151, n_B_ = 146	*271 (102–683)	*253 (103–669)	0.1094*†
Distal thoracic aorta	436 (173–725	450 (56–776)	0.7938†
n_A_ = 151, n_B_ = 146	*442 (175–735)	*422 (53–728)	0.0177*†

Data are medians and ranges in parentheses. n_A_ = number of vessels in group A (iopromide 370); n_B_ = number of vessels in group B (iomeprol 400).

* Attenuation in the great arteries was standardized with an iodine delivery rate of 1.5 g I/s.

† Two-group Wilcoxon rank sum test (two-sided).

The consistency of contrast enhancement calculated by ROI differences between the proximal ascending aorta and distal thoracic aorta was not significantly different between the two groups. The average consistency of contrast enhancement for iopromide 370 and iomeprol 400 was 49 ± 47 HU and 55 ± 56 HU, respectively (*P* = 0.12).

### Evaluation of streak artifacts in right atrium

Nine of 151 patients (6%) in group A and 7 of 146 patients in group B (5%) showed a mild streak artifact. No case showed moderate or severe streak artifacts that obscured the right coronary artery.

### Change of heart rate and adverse effects with injection of contrast material

There was no moderate or severe adverse reaction to the intravenous contrast agent, but 13 patients had a mild adverse reaction such as nausea (*n* = 6), dizziness (*n* = 3), and urticaria (*n* = 4). Group A had eight cases (5.3%) with mild adverse reactions (three cases of nausea, one case of dizziness, four cases of urticaria) and group B had five cases (3.4%) with mild adverse reactions (three cases of nausea, two cases of dizziness). There was no significant difference in the frequencies of adverse reactions to the contrast agents between the two groups (*P* = 0.43). All of the patients’ symptoms were resolved soon after conservative treatment.

The average change in the heart rate after injection of contrast material was 2.9 beats per minute (median, 2; range, 0—20) for group A and 3.2 (median, 3; range, 0—25) for group B, respectively. For group A, 105 patients showed a decrease or no change in heart rate after injection of contrast material, and 41 patients showed increased heart rates (mean, 3.1; median, 3; range, 1—20 beats per minute). For group B, 111 patients showed a decrease or no change in heart rates after injection of contrast material and 35 patients showed an increased heart rate (mean, 3.1; median, 2; range, 1—10 beats per minute).

## Discussion

An optimal contrast agent application protocol for CCTA is critical as the ability to diagnose coronary artery disease mainly depends on adequate visualization of the coronary arteries. Arterial enhancement is generally determined by the number of iodine molecules administered. The rate of iodine administration can be increased either by increasing the injection flow rate or by increasing the iodine concentration of the contrast agent (11). However, the degree of arterial enhancement following the intravenous injection of the same amount and type of contrast material is highly variable among individuals for physiological parameters such as cardiac output and central blood volume (11). Moreover, according to Cademartiri et al. (12), contrast bolus geometry may not only depend on contrast density and flow but also on contrast volume, bolus chaser, and heart diseases. In our study, we strictly controlled the factors that could influence the contrast geometry, such as the total volume and injection rate of the contrast material as well as body weight.

Several investigators have reported a major impact of different iodine fluxes on arterial enhancement (7, 10, 13, 14). The arterial enhancement is proportional to the iodine flux; the higher iodine flux, the higher the arterial enhancement. The iodine flux can be increased by increasing the iodine concentration or injection rate (10). Slightly higher attenuation values found for iomeprol 400 can be attributed to the higher iodine delivery rate (1.60 g I/s) as compared with that of iopromide 370 (1.48 g I/s). However, the standardized attenuation values for an iodine flux of 1.5 g I/s were shown to be significantly higher for the use of iopromide 370 as compared with iomeprol 400 in the proximal ascending aorta, thoracic descending aorta, and the average value across the great arteries. This advantage was also found in the RCA, LM, and LCX and the average across the coronary arteries. The higher values for the use of iopromide 370 after standardization of the iodine flux may reflect the effects of the different viscosities of the two contrast agents (12.6 mPas for iomeprol 400 and 9.5 mPas for iopromide 370 at 37°C). The higher viscosity of iomeprol 400 might result in inhomogeneous mixing and therefore a non-superior attenuation value, which seemed to be compensated by the higher iodine flux. When the viscosity of the contrast agent approaches that of blood, mixing of the two fluids takes place more easily. This is of importance in cardiovascular examinations (15).

As a limitation of this study, we did not evaluate vessels with atherosclerotic disease with extensive calcification, because these factors may affect the visualization and attenuation of vessels. We administered a total amount of 70 ml of contrast material at a flow rate of 4 ml/s regardless of the BMI and height of patients. However, the BMI did not differ significantly in the two groups; therefore no bias in the comparison between the groups was expected.

In conclusion, the image quality of CCTA using the same amount of iopromide 370 or iomeprol 400 at the same injection rate is similarly excellent with different iodine fluxes. With standardization for an iodine flux, the attenuation is significantly higher when using iopromide 370.
